# An Evaluation of the Adequacy of the Liberal Transfusion Strategy in Endoscopy-Assisted Metopic, Coronal, or Sagittal Craniosynostosis Surgeries: A Retrospective Observational Study

**DOI:** 10.3390/medicina61040618

**Published:** 2025-03-28

**Authors:** Turan Evran, Emrah Egemen, Barış Albuz, İsmet Çopur, Rasim Asar, Aslı Mete Yıldız, Seher İlhan, Serkan Civlan, Hülya Sungurtekin

**Affiliations:** 1 Department of Anesthesiology and Reanimation, Faculty of Medicine, Pamukkale University, Denizli 20160, Turkey; icopur@pau.edu.tr (İ.Ç.); amete@pau.edu.tr (A.M.Y.); seheri@pau.edu.tr (S.İ.); hsungurtekin@yahoo.com (H.S.); 2 Department of Neurosurgery, Faculty of Medicine, Pamukkale University, Denizli 20160, Turkey; eegemen@pau.edu.tr (E.E.); barisa@pau.edu.tr (B.A.); rasar@pau.edu.tr (R.A.); scivlan@pau.edu.tr (S.C.)

**Keywords:** blood loss, blood management, craniosynostosis, endoscopic surgery, transfusion

## Abstract

*Background and Objectives*: This study aims to evaluate the adequacy of the liberal transfusion strategy applied in patients undergoing endoscopy-assisted Metopic, Coronal, or Sagittal craniosynostosis surgery according to the Pre-Transfusion and Post-Transfusion Estimated Red Blood Cell Mass (ERCM) ratios. *Materials and Methods*: This retrospective cohort study, conducted at the Pamukkale University Faculty of Medicine (2017–2023), utilized anesthesia, surgical records, and hospital electronic data of patients undergoing endoscopic craniosynostosis surgery. The primary endpoints were the rates of Post-Transfusion 1st-hour ERCM/Pre-Transfusion ERCM (%) and Post-Transfusion 24th-hour ERCM/Pre-Transfusion ERCM (%). The secondary endpoints were determined as Hemoglobin (Hb) and Hematocrit (Hct) values at the 1st and 24th hours after surgery, Calculated Blood Loss (CBL) during surgery (%), total 24 h CBL (%), ERCM (%), and Estimated Blood Loss (EBV) during surgery and total 24 h transfusions, Packed Red Blood Cells (PRBCs) (mL/kg) amounts during surgery, and total 24 h transfusions. *Results*: A total of 86 pediatric craniosynostosis cases were evaluated and categorized into Metopic (*n* = 38), Sagittal (*n* = 33), and Coronal (*n* = 15) groups, with Post-Transfusion evaluation conducted across these groups. Post-Transfusion 1st-hour ERCM/Pre-Transfusion ERCM ratios were found to have median values of 90.70% in the Metopic group, 91.61% in the Sagittal group, and 93.09% in the Coronal group. Post-Transfusion 24th-hour ERCM/Pre-Transfusion ERCM ratios were found to be median values of 94.05% in the Metopic group, 88.3% in the Sagittal group, and 87.08% in the Coronal group. *Conclusions*: The liberal transfusion strategy provided adequate transfusion, maintaining ERCM ratios within the 85–115% range across all groups. Significant decreases in Hb and Hct levels were observed from preoperative to postoperative measurements at 1 and 24 h. Changes in CBL, ERCM, EBV, and PRBC volumes were noted between the postoperative 1 h and 24 h measurements across all groups.

## 1. Introduction

Craniosynostosis, a congenital anomaly characterized by the premature closure of one or more cranial sutures, restricts brain development and causes abnormal head shapes [1]. While often isolated, it may be associated with genetic syndromes. Diagnosis involves clinical evaluation, physical examination, and imaging, with surgical intervention being the only treatment to prevent intracranial pressure and cosmetic issues [2]. Open cranial vault surgery, commonly performed between 6 and 12 months, is effective but associated with prolonged recovery and significant blood loss [3]. Alternatively, endoscopy-assisted craniosynostosis surgery (EACS) combined with helmet therapy, typically performed before 6 months of age, offers reduced bleeding and shorter recovery times [4].

A critical challenge in craniosynostosis surgery is managing fluid and blood transfusion due to the significant blood loss associated with the large cranial structure in pediatric patients [5]. The accurate assessment of blood loss remains difficult, often leading to risks from under- or over-transfusion [6]. Current transfusion practices rely on Estimated Blood Loss (EBL) and Hemoglobin (Hb) levels, with guidelines recommending minimum intraoperative Hb levels of 8–10 g/dL, to prevent hypoxia and complications [7,8,9]. The European Society of Anaesthesiology and Intensive Care suggests target Hb levels of 7–9 g/dL for intraoperative bleeding, excluding premature infants and cyanotic neonates [10]. However, standard Hb threshold values have not yet been specified regarding transfusion protocol and management in pediatric neurosurgery [11].

Although more studies are needed to determine the ideal Hb level in pediatric patients, recent research suggests that avoiding unnecessary transfusions for non-ongoing bleeding in pediatric surgery is appropriate and highlights the need for individualized transfusion decisions based on physiological conditions rather than fixed Hb thresholds [12,13]. Proposed target Hb levels in pediatric neurosurgical patients vary, with recommendations such as 9.5 g/dL for transfusion and acceptable changes in Estimated Red Blood Cell Mass (ERCM) ranging from 12% to 15% to guide transfusion appropriateness [13,14,15].

This study evaluates the adequacy of liberal transfusion strategies in patients undergoing EACS for Metopic, Coronal, or Sagittal craniosynostosis using Pre- and Post-Transfusion ERCM ratios. We hypothesize that while transfusion adequacy will be maintained across surgical subtypes, there may be an increased rate of excessive transfusions.

## 2. Materials and Methods

This retrospective cohort study was conducted at Pamukkale University’s Faculty of Medicine between 2017 and 2023 using anesthesia and surgical records of patients undergoing EACS and information obtained from hospital electronic data systems. This study was approved by the Ethics Committee of Non-Interventional Clinical Trials of Pamukkale University (Date and Number of Approval: 10 January 2024, E.474199) and was carried out in accordance with the Declaration of Helsinki.

### 2.1. Data Collection

Patients who underwent EACS were divided into three groups: Metopic, Coronal, and Sagittal, according to the type of closed suture. Gender, age (day), weight (kg), and American Society of Anesthesiologists (ASA) score were recorded for the patients in each group. The amount of fluid they received during the intraoperative (Intra-op) period was recorded in milliliters (mL), and the duration of anesthesia and surgery was recorded in minutes (min). The duration of anesthesia was defined as the time between anesthesia induction and the patient’s awakening, while the surgical time was defined as the time between the start of the surgical incision and the final closing suture. The duration of each patient’s stay in the intensive care unit was recorded in hours, and their stay in the ward and the hospital was recorded as days. It was also recorded whether the patients experienced death during their hospitalization. The Hb and Hematocrit (Hct) values of all patients at preoperative (Pre-op) and postoperative (Post-op) 1 and 24 h were recorded. Blood samples taken within the first hour of each patient’s admission to Post-op intensive care, and the first blood samples taken on the next day of surgery were, respectively, “Post-op 1. the hour” and “Post-op 24. the watch was described as having” values of. The amount of allogeneic Packed Red Blood Cells (PRBCs) transfused into the patients in the intraoperative period, postoperative period, and the first 24 h were recorded in mL.

### 2.2. Inclusion Criteria

Patients under one year of age who underwent EACS, underwent allogeneic PRBC transfusion during the Intra-op period, and underwent standard anesthesia and surgical protocols were included in this study.

### 2.3. Exclusion Criteria

Patients who had a known blood transfusion before surgery; those with a syndrome; those who had more than one closed suture line; those whom another surgeon operated on; those who were re-operated; those who had an abnormal International Normalized Ratio, Prothrombin Time, or Activated Partial Thromboplastin Time value during the Pre-op period; those with a platelet count below 100,000/mm^3^, known bleeding history, or abnormal electrolyte imbalance in Sodium (mEq/L), Potassium (mEq/L), Calcium (mg/dL or mmol/L), and Chloride (mEq/L) levels; and those known to be taking nonsteroidal anti-inflammatory drugs that may affect the bleeding profile prior to surgery were excluded from this study.

### 2.4. Surgical Technique

EACS was preferred for all synostosis types in our study. A single surgeon performed all the surgeries. Patients with Metopic suture synostosis were generally taken to the operating table in a supine and neutral position. A transverse skin incision of approximately 3 cm was made at the anterior border of the hairline. After a burr hole was placed on the 1 cm lateral to the Metopic suture, craniectomy was performed by crossing the middle line with the help of a rongeur. The subcutaneous and epidural areas were dissected with the help of an endoscope. Craniectomy was performed first posteriorly to bregma, then to the anterior interorbital, extending to an approximately 1–2 cm triangular suturectomy. During craniectomy, the nasofrontal suture was preserved in the anterior, and the procedure was performed up to the nasofrontal suture.

Patients with Sagittal suture synostosis were usually taken to the operating table in the prone position with a slight head extension (Sphinx position). However, a supine full lateral position was also preferred in some patients. Depending on the surgeon’s preference, a single or two incisions were performed along the Sagittal suture. After placing the burr hole 1 cm lateral to the Sagittal suture, craniectomy was performed by crossing the suture with the help of a rongeur. The emissary’s veins were cauterized using an endoscope, and a craniectomy was performed along the suture line. The craniectomy width was determined as 2–4 cm. Bregma in the anterior and lambda in the posterior were accepted as the limits of suturectomy.

Patients with Coronal suture synostosis were taken to the operating table with their heads turned to the side and contralateral in the supine position. A linear skin incision of approximately 3 cm in length was made parallel to the Coronal suture to center the Stephanion point. The craniectomy width was between 1 and 2 cm. Bleeding from the bone at the pterion level was carefully stopped with the help of an endoscope. The suturectomy boundaries were accepted as being between the bregma and the pterion point.

### 2.5. Anesthesia Management

All surgical procedures were performed under general anesthesia. Standard monitoring included electrocardiography, invasive arterial blood pressure, capnography, pulse oximetry, body temperature measurement with a skin probe, and central venous pressure monitoring with the help of a central venous catheter. A heating blanket was applied to all patients to prevent heat loss during the Intra-op period. Anesthesia induction was achieved using propofol (2.0 mg/kg) and rocuronium (0.6 mg/kg). Anesthesia was maintained with a sevoflurane and remifentanil infusion of 0.1–0.2 µg/kg/min. In order to reduce the amount of bleeding, tranexamic acid (10 mg/kg loading, followed by 5 mg/kg maintenance) was started after intubation and given to all patients during the anesthesia period. Tranexamic acid was not given to the patients in the Post-op period. Autologous transfusion, Pre-op hemodilution, and blood preservative techniques or devices were not used in any of the patients.

A balanced crystalloid solution (2.5% dextrose and 0.45% isotonic saline) was administered as a continuous infusion for maintenance fluid therapy. According to the Pre-op fasting period, Intra-op fluid losses, and basal fluid requirements, the amount of fluid was determined by the anesthesiologist who followed the patient during the operation. Allogeneic PRBC transfusion was performed in all patients during the Intra-op period.

Although there is no standard protocol for transfusion in EACS, allogenic PRBC transfusion is routinely performed with the joint approval of the anesthesia and surgical teams if the Hb level drops below 9 g/dL or is expected in cases of ongoing bleeding in our clinic, and a Hb level of 10 g/dL and above is targeted after transfusion.

At the end of the surgery, paracetamol 15 mg/kg intravenous infusion was given to all patients to provide Post-op analgesia. All patients were extubated in the operating room.

Post-Transfusion in our study 1st-hour ERCM/Pre-Transfusion ERCM and Post-Transfusion 24th-hour ERCM/Pre-Transfusion ERCM rates are below 85% “insufficient transfusion”, 85–115% among them, it was defined as “sufficient transfusion” and over 115% as “excessive transfusion”. This calculation method has been used to evaluate transfusion adequacy in previous studies [14,15]. In addition, the Calculated Blood Loss (CBL) percentage, Transfusion ERCM (%), and transfusion Estimated Blood Volume (EBV) (%) were calculated for each patient according to the methods used by Lopez et al. [16]. The Hct value of the PRBCs used in our hospital was taken as 70(%). Perioperative Transfusion Volumes, Percentages, and Blood Loss Calculations are shown in Table 1.

The primary endpoints were the rates of Post-Transfusion 1st-hour ERCM/Pre-Transfusion ERCM/and Post-Transfusion 24th-hour ERCM/Pre-Transfusion ERCM (%)/Pre-Transfusion ERCM. The secondary endpoints were determined as Hb and Hct values at the 1st and 24th hours after surgery; CBL during surgery (%); total 24 h CBL (%); ERCM (%); EBV during surgery and total 24 h transfusions; and PRBC (mL/kg) amounts during surgery and total 24 h transfusions.

### 2.6. Statistical Analysis

For the power analysis of this study, G*Power version 3.1.9.7. was used. Craniosynostosis cases with a prevalence of 0.02% were used to calculate 95% validity and 95% reliability for the patients to be included in this study. It was determined that a total of 36 patients would be sufficient. The collected data were analyzed using the IBM Statistical Package for the Social Sciences 25.0 software. The Chi-square test was used to compare the initial characteristics of nominal variables. The Mann–Whitney U test was used to analyze continuous variables between the groups. The Wilcoxon test was used to compare the transfusion percentages between the groups. The numerical variables obtained were written as Mean ± SD (Median). A value of *p* < 0.05 was considered statistically significant in all analyses.

## 3. Results

**Figure 1:**
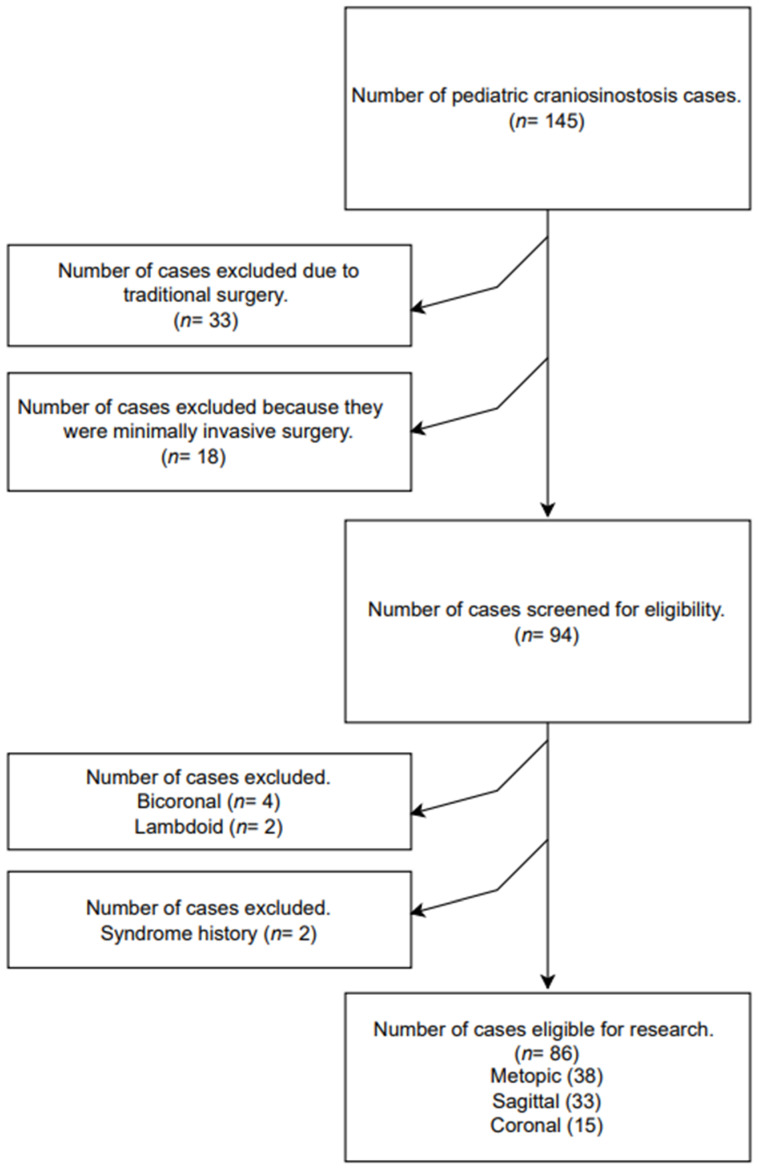
Flowchart.

A total of 145 pediatric cases of craniosynostosis were evaluated in this study. In total, 33 patients who underwent open surgery and 18 who underwent minimally invasive surgery were excluded from this study. Out of 94 patients, 4 bi-Coronal, 2 lambdoid, and 2 syndromic patients were excluded, and a total of 86 patients were included in this study. The patients were divided into three groups: Metopic (*n* = 38), Sagittal (*n* = 33), and Coronal (*n* = 15) (Figure 1).

Our study examined the demographic and perioperative parameters in the Metopic, Sagittal, and Coronal groups (Table 2). In the Metopic group, 23.7% were female and 76.3% were male. In the Sagittal group, 21.2% were female and 78.8% were male. In the Coronal group, 46.7% were female and 53.3% were male. The mean age was 93.29 ± 17.98 days in the Metopic group, 89.61 ± 16.71 days in the Sagittal group, and 98.93 ± 21.26 days in the Coronal group. The mean weight was 5.47 ± 1.19 kg in the Metopic group, 5.32 ± 1.09 kg in the Sagittal group, and 5.47 ± 1.28 kg in the Coronal group.

ASA scores were found to be mostly ASA 1 with 94.7(%) in the Metopic group, ASA 1 with 84.8(%) in the Sagittal group, and ASA 1 with 80(%) in the Coronal group.

The mean duration of surgery was 58.89 ± 17.16 min in the Metopic group, 67.88 ± 32.26 min in the Sagittal group, and 57 ± 9.02 min in the Coronal group. The mean duration of general anesthesia was 182.89 ± 52.37 min in the Metopic group, 176.52 ± 46.85 min in the Sagittal group, and 168.33 ± 37.78 min in the Coronal group.

Intra-hospital death was observed in 1.36(%) of all patients, while no mortality was observed in the Coronal group.

The median of the delivered liquid amounts is 50.00 mL for Metopic, 55.00 mL for Sagittal, and 45.00 mL for Coronal. There was no statistical difference between age, weight, ASA score, the duration of surgery, the duration of general anesthesia, the amount of fluids given, the duration of intensive care, and hospital stay between all groups (*p* > 0.05). During the Intra-op period, the median is 50 mL in each group (range 40–60 mL). In the Post-op period, the median values are 22.5 mL (range 0–51.25 mL) for Metopic, 30 mL (range 0–50 mL) for Sagittal, and 30 mL (range 0–40 mL) for Coronal. The total transfusion amounts for the first 24th hour were measured as median values of 75 mL for Metopic and Coronal and 80 mL for Sagittal.

There was no statistically significant difference between the groups according to Intra-op, Post-op, and total first 24th-hour Transfusion PRBC amounts (*p* > 0.05).

In our study, Post-Transfusion evaluation was performed between the Metopic, Sagittal, and Coronal groups. Within the group, the Post-Transfusion 1st-hour ERCM/Pre-Transfusion ERCM and Post-Transfusion 24th-hour Hourly ERCM/Pre-Transfusion ERCM ratios were evaluated (Table 3). The Post-Transfusion 1st-hour ERCM/Pre-Transfusion ERCM Ratios were found to have median values of 90.70(%) (93.6 ± 14.1) in the Metopic group, 91.61(%) (93.96 ± 14.84) in the Sagittal group, and 93.09(%) (97.28 ± 18.1) in the Coronal group.

The percentage of patients with an ERCM/Pre-Transfusion ERCM below 85(%) at the Post-Transfusion 1st-hour was 26.3(%) in the Metopic group, 25.2(%) in the Sagittal group, and 26.7(%) in the Coronal group. The ERCM/Preoperative ERCM at the 1st hour after surgery was between 85% and 115%; the percentage of patients was 63.2% in the Metopic group, 69.7% in the Sagittal group, and 60% in the Coronal group. The percentage of patients with a Post-op 1st hour ERCM/Pre-op ERCM above 115% was recorded as 10.5% in the Metopic group, 6.1% in the Sagittal group, and 13.2% in the Coronal group.

There was no significant difference between the ERCM/Pre-Transfusion ERCM Ratios between the groups and within the group at the Post-Transfusion 1st-hour (*p* = 0.921).

Post-Transfusion 24th-hour ERCM/Pre-Transfusion ERCM Ratios were found to be Median values of 94.05(%) (94.36 ± 12.67) in the Metopic group, 88.3(%) (90.45 ± 18.28) in the Sagittal group, and 87.08(%) (91.69 ± 18.1) in the Coronal group.

The percentage of patients with an ERCM/Pre-Transfusion ERCM below 85% at the Post-Transfusion 24th-hour was found to be 18.4% in the Metopic group, 36.4% in the Sagittal group, and 20% in the Coronal group. The percentage of patients with a Post-Transfusion 24th-hour ERCM/Pre-Transfusion ERCM between 85% and 115% was found to be 78.9% in the Metopic group, 54.5% in the Sagittal group, and 73.3% in the Coronal group. 

There was no significant difference between the ERCM/Pre-Transfusion ERCM Ratios between the groups and within the group at the Post-Transfusion 24th-hour (*p* = 0.255).

Our study showed no difference between the groups when Pre-op Hb and Hct values were compared in the Metopic, Sagittal, and Coronal groups at the 1st hour and 24th hour Post-op (*p* > 0.05) (Table 4).

In the Metopic groups, the decrease in Hb levels at the 1st hour Post-op (10.25 ± 1.56) and 24th hour Post-op (10.18 ± 1.14) was statistically significant (*p* = 0.002). In Sagittal groups, the decrease in Hb levels at the 1st hour Post-op (10.14 ± 1.19) and the 24th hour Post-op (9.9 ± 1.78) was statistically significant (*p* = 0.001). In the Coronal group, there was no significant decrease at 1 h Post-op (*p* = 0.222), and the decrease in Hb levels at 24 h (9.88 ± 1.68) was significant (*p* = 0.020).

The decrease in Hct values between the 1st hour Post-op (29.88 ± 3.96) and the 24th hour Post-op (30.08 ± 3.36) in the Metopic groups was statistically significant (*p* = 0.005, *p* = 0.007). The decrease in Hct values between the 1st hour Post-op (30.18 ± 3.34) and the 24th hour Post-op (29.02 ± 4.98) in the Sagittal groups was statistically significant (*p* = 0.001, *p* = 0.003, respectively). In the Coronal group, while no significance was found at the 1st hour Post-op (*p* = 0.233), the decrease in Hct levels at 24 h (29.13 ± 4.49) was significant (*p* = 0.011).

Our study compared Intra-op and total first 24 h CBL, Transfusion ERCM, transfusion amounts, and Transfusion EBV between the Metopic, Sagittal, and Coronal groups (Table 5).

The median values of CBL in the Intra-op period were found to be 34.87% (32.81 ± 14.08) in the Metopic group, 33.66% (31.81 ± 17.3) in the Sagittal group, and 29.72% (29.75 ± 15.63) in the Coronal group. In the first 24 h, these rates increased to 45.96% (45.94 ± 14.97) in the Metopic group, 51.3% (51.3 ± 23.78) in the Sagittal group, and 52.25% (47.21 ± 19.49) in the Coronal group. These increases were statistically significant in the Metopic (*p* < 0.001), Sagittal (*p* < 0.001), and Coronal (*p* = 0.005) groups.

The Intra-op Transfusion ERCM values were measured as 27.14% (26.46 ± 6.94) in the Metopic group, 25.48% (27.77 ± 6.7) in the Sagittal group, and 25.7% (27.03 ± 6.84) in the Coronal group. Total First 24 h Transfusion ERCM was determined to be 52.5% (54.8 ± 16.98) in the Metopic 56% (56.68 ± 19.06) in the Sagittal group, and 52.5% (53.67 ± 16.08) in the Coronal group. These increases were statistically significant in the Metopic (*p* < 0.001), Sagittal (*p* < 0.001), and Coronal (*p* = 0.001) groups.

Transfusion PRBC amounts in ml/kg were found to be 10 (9.62 ± 2.39) in the Metopic group, 9.52 (9.5 ± 2.88) in the Sagittal group, and 10 (9.6 ± 2.39) in the Coronal group during the Intra-op period. During the first 24 h, these values were found to be 13.33 (14.57 ± 4.47) in the Metopic group, 15 (15.47 ± 5.22) in the Sagittal group, and 14 (14.11 ± 3.23) in the Coronal group. These increases were statistically significant in the Metopic (*p* < 0.001), Sagittal (*p* < 0.001), and Coronal (*p* = 0.004) groups.

The Intra-op Transfusion EBV was measured as 12.5% in the Metopic and Coronal groups and 11.9% (11.87 ± 3.6) in the Sagittal group. The Transfusion EBV values were found to be 16.49% (18.3 ± 5.41) in the Metopic group, 18.15% (19.19 ± 6.55) in the Sagittal group, and 18.33% (18.3 ± 3.87) in the Coronal group in the first 24 h. These increases were statistically significant in the Metopic (*p* < 0.001), Sagittal (*p* < 0.001), and Coronal (*p* = 0.003) groups.

## 4. Discussion

Our study evaluated the adequacy of a liberal transfusion strategy in patients undergoing Metopic, Coronal, and Sagittal endoscopic craniosynostosis surgery using Pre- and Post-Transfusion ERCM ratios. The Median Post-Transfusion ERCM/Pre-Transfusion ERCM values were within the adequate transfusion range (85–115%) across all groups: Metopic (90.7% at 1 h, 94.1% at 24 h), Sagittal (91.6% at 1 h, 88.3% at 24 h), and Coronal (93.1% at 1 h, 87.1% at 24 h). Most patients received adequate transfusions (1st hour: Metopic 63.2%, Sagittal 69.7%, Coronal 60%; 24th hour: Metopic 78.7%, Sagittal 54.5%, Coronal 73.3%). The proportion of patients receiving insufficient transfusions was higher than those receiving excessive transfusions across all groups. At the 1st postoperative hour, insufficient transfusions were observed in 26.3% of the Metopic group, 25.2% of the Sagittal group, and 26.7% of the Coronal group. By the 24th hour, these rates shifted to 18.4%, 36.4%, and 20%, respectively. In contrast, excessive transfusions were the least common, occurring in 10.5% (Metopic), 6.1% (Sagittal), and 13.2% (Coronal) of patients at the 1st hour and decreasing to 2.6%, 9.1%, and 6.7%, respectively, at the 24th hour. However, contrary to our hypothesis, insufficient transfusions exceeded excessive transfusions, likely due to a transfusion trigger of Hb < 9 g/dL compared to studies using Hct < 30% as a threshold.

Statistically significant reductions in Hemoglobin (Hb) and Hematocrit (Hct) levels were observed in the Metopic and Sagittal groups at both 1 h and 24 h postoperatively, while the Coronal group exhibited significant decreases at the 24 h mark. Additionally, the intraoperative blood loss percentage (CBL%) increased significantly across all groups within the first 24 h. The percentage of transfused estimated red cell mass (ERCM) also showed a notable increase in all groups by the 24 h mark. Correspondingly, intraoperative transfusion volumes, including Packed Red Blood Cells (PRBCs) and EBV, demonstrated significant increases across all groups within the same timeframe.

In perioperative transfusion management for patients undergoing craniosynostosis surgery, many studies have relied on EBV, ERCM, and EBL calculations [17,18,19]. However, recent research has moved away from using these calculations to assess transfusion adequacy. Older studies evaluated transfusion adequacy based on Pre-Transfusion/Post-Transfusion ERCM exchange rates. Kearney et al. reported that while 70% of intraoperative transfusions were managed correctly, only 29% were truly appropriate, leaving the majority of postoperative transfusions unnecessary [14]. Similarly, Kang et al. analyzed 43 patients who underwent primary craniosynostosis repair and found that, based on Pre-Transfusion/Post-Transfusion ERCM ratios, 18% received excessive transfusions, 70% had appropriate transfusions, and 12% were under-transfused [15]. In our study, adequate transfusion rates exceeded 60% across all groups, highlighting the effectiveness of our approach. However, the higher rates of excessive transfusion indicate a need for further optimization. The significant postoperative decrease in Hb and Hct levels, coupled with increased intraoperative blood loss (CBL%) and higher transfusion volumes (PRBC and EBV) within 24 h, underscores the critical importance of precise transfusion strategies.

Meier et al. reported an EBL of approximately 25% of the EBV during endoscopic procedures [20]. Similarly, Han et al. documented a median intraoperative transfusion volume of 36 mL in 140 patients with non-syndromic craniosynostosis, with an EBL-to-EBV ratio of 7.5% ± 5.3% [21].

The higher Calculated Blood Loss observed in our study compared to studies reporting 25% and 7.5% EBL-to-EBV ratios, respectively, may stem from our reliance on Calculated Blood Loss methods, which provide greater accuracy than subjective surgical estimates [20,21]. Some studies show that the Calculated Blood Loss method provides more accurate results than the Estimated Blood Loss of the surgical team in different surgical and age groups [7,16,22]. The literature also supports the idea that bleeding varies between surgeons in the same surgical group [23].

Sakar et al. documented a median total intraoperative and postoperative transfusion volume of 32 mL in a cohort of 31 patients [24]. Furthermore, Soobey et al. noted an average intraoperative blood transfusion volume of 3 mL/kg in 49 single-suture craniosynostosis cases, with postoperative Hematocrit values averaging 25.3% ± 3.3% and with an intraoperative crystalloid fluid use of 34 mL/kg [25]. Notably, our liberal transfusion strategy, with a trigger of Hb < 9 g/dL and a target of Hb 10 g/dL, likely contributed to higher transfusion volumes and reduced crystalloid fluid use compared to restrictive strategies reported in the literature.

Within the scope of this strategy, we think that determining the transfusion trigger threshold as Hb 9 g/dL and the target value as Hb 10 g/dL is effective in performing more PRBC transfusions and using less crystalloid fluid.

Our study’s strengths include the use of objective ERCM ratios, a homogeneous patient cohort, and a single-center, single-surgeon design, which minimizes variability and enhances data reliability. However, limitations include its retrospective design, single-center scope, and unequal subgroup sizes, which reduce generalizability and subgroup analysis strength. Variability in PRBC Hct values across institutions and the absence of a restrictive transfusion comparator group further limit this study’s applicability. Additionally, the lack of advanced techniques to reduce perioperative transfusion, such as blood recovery systems, may have influenced outcomes. In this study, transfusion-related clinical outcomes were not evaluated. To accurately assess transfusion-related clinical outcomes, it is essential to first demonstrate that sufficient and necessary transfusions have been performed.

Future studies should explore the use of laboratory and imaging methods to assess organ perfusion, adopt alternative transfusion thresholds, and evaluate blood conservation techniques, such as intraoperative blood recovery systems and pharmacologic interventions. Multi-center, prospective trials with larger patient cohorts are essential to validate and optimize transfusion protocols for endoscopic craniosynostosis surgery. These efforts will enable more individualized transfusion strategies that balance patient safety with resource utilization, ensuring optimal outcomes in this delicate pediatric population.

## 5. Conclusions

Using a liberal transfusion strategy, we observed that the Post-Transfusion 1st-hour and 24th-hour ERCM/Pre-Transfusion ERCM ratios remained within the 85–115% adequacy range. In intra-group analyses, most patients received adequate transfusions, followed by those with insufficient transfusions, while excessive transfusions were the least common. These patterns were consistent across all surgical groups. Our findings indicate that the liberal transfusion strategy effectively ensures adequate transfusion. However, the volume of transfusions in our study exceeded those reported in the literature, underscoring the need for more individualized, patient-centered approaches to refine transfusion decisions and improve their adequacy.

## Figures and Tables

**Table 1:** Perioperative Transfusion Volumes, Percentages, and Blood Loss Calculations.

Parameter	Calculation Method
Intra-op Transfusion PRBC (mL/kg)	Intra-op transfused PRBC (mL)/Body Weight (kg)
Post-op Transfusion PRBC (mL/kg)	Post-op transfused PRBC (mL)/Body Weight (kg)
Total 24 h Transfusion PRBC (mL/kg)	Intra-op transfused PRBC (mL/kg) + Post-op transfused PRBC (mL/kg)
Pre-op EBV (mL)	Body Weight (kg) × 80 mL/kg
Pre-Transfusion ERCM (mL)	Pre-op EBV × Hct (%)/100
Post-Transfusion 1st-hour ERCM/Pre-Transfusion ERCM	(Pre-op EBV × Post-op 1st-hour Hct (%)/100)/(Pre-op EBV × Pre-op Hct (%)/100)
Post-Transfusion 24th-hour ERCM/Pre-Transfusion ERCM	(Pre-op EBV × Post-op 24th-hour Hct (%)/100)/(Pre-op EBV × Pre-op Hct (%)/100)
Intra-op Transfusion ERCM (mL)	Intra-op Transfusion (PRBC) (mL) × 70/100
Post-op Transfusion ERCM (mL)	Post-op Transfusion PRBC (mL) × 70/100
Total 24 h Transfusion ERCM (mL)	Intra-op Transfusion ERCM + Post-op Transfusion ERCM
Intra-op Transfusion ERCM (%)	(Intra-op Transfusion ERCM/Pre-Transfusion ERCM) × 100
Total 24 h Transfusion ERCM (%)	(Intra-op Transfusion ERCM + Post-op Transfusion ERCM)/Pre-Transfusion ERCM × 100
Intra-op CBL (%)	(Pre-Transfusion ERCM + Intra-op Transfusion ERCM − Post-Transfusion 1st-hour ERCM)/Pre-Transfusion ERCM × 100
Total 24 h CBL (%)	(Pre-Transfusion ERCM + Total 24 h Transfusion ERCM − Post-Transfusion 24th-hour ERCM)/Pre-Transfusion ERCM × 100
Intra-op Transfusion EBV (%)	(Intra-op Transfusion PRBC (mL)/Pre-op EBV) × 100
Total 24 h Transfusion EBV (%)	(Intra-op Transfusion PRBC (mL) + Post-op Transfusion PRBC (mL))/(Pre-op EBV) × 100

PRBC (Packed Red Blood Cells); EBV (Estimated Blood Volume); ERCM (Estimated Red Blood Cell Mass); CBL (Calculated Blood Loss).

**Table 2:** Demographic and perioperative parameters according to Metopic, Sagittal, and Coronal groups.

		Metopic (n/%) (Mean ± SD)	Sagittal (n/%) (Mean ± SD)	Coronal (n/%) (Mean ± SD)	*p*-Value
Gender	Female	9 (23.7)	7 (21.2)	7 (46.7)	0 .154
	Male	29 (76.3)	26 (78.8)	8 (53.3)	
ASA Score	1	36/94.7	28/84.8	12/80.0	0 .232
	2	2/5.3	5/15.2	3/20.0	
Mortality	exitus	1/2.6	1/3.0	0/0.0	0 .801
	No	37/97.4	32/97.0	15/100.0	
Age (day)		93.29 ± 17.98	89.61 ± 16.71	98.93 ± 21.26	0.414
Weight (kg) (Median)		5.47 ± 1.19 (5.00)	5.32 ± 1.09 (5.2)	5.47 ± 1.28 (5.1)	0.885
Duration of Surgery (min) (Median)		58.89 ± 17.16 (55)	67.88 ± 32.26 (55)	57 ± 9.02 (55)	0.976
Duration of General Anesthesia (min) (Median)		182.89 ± 52.37 (175)	176.52 ± 46.85 (170)	168.33 ± 37.78 (180)	0.775
Given Liquid mL (Median)		55 ± 25.39 (50)	61.67 ± 32.71 (55)	53.07 ± 18.87 (45)	0.875
İntra-op Transfusion PRBC (Median)		51.32 ± 14.69 (50)	49.85 ± 16.61 (50)	52.67 ± 13.87 (50)	0.642
Post-op Transfusion PRBC (Median)		27.5 ± 27.82 (22.5)	30.21 ± 27.37 (30)	26.33 ± 23.64 (30)	0.753
Total Transfusion PRBC (Median)		78.29 ± 24.25 (75)	80.97 ± 27.22 (80)	76.67 ± 22.96 (75)	0.842
Duration of Stay in Intensive Care (hours) (Median)		14.58 ± 8.58 (12)	14.55 ± 6.58 (12)	21 ± 28.2 (13)	0.892
Duration of Hospital Stay (days) (Median)		3.5 ± 1.78 (3)	3.73 ± 1.77 (3)	3.87 ± 2.53 (3)	0.758

ASA (American Society of Anesthesiologists); PRBC (Packed Red Blood Cells).

**Table 3:** Post-Transfusion in Metopic, Sagittal, and Coronal Groups 1st-hour ERCM/Pre-Transfusion ERCM vs Post-Transfusion 24th-hour ERCM/Comparison of Pre-Transfusion ERCM Rates.

		Metopic	Sagittal	Coronal	*p*
Post-Transfusion 1st-hour ERCM/ Pre-Transfusion ERCM (%)	Med (IQR)	93.6 ± 14.1 (90.7)	93.96 ± 14.84 (91.61)	97.28 ± 18.18 (93.09)	0.925
	Under 85	10 (26.3)	8 (25.2)	4 (26.7)	0.921
	Range 85–115	24 (63.2)	23 (69.7)	9 (60)	
	Above 115	4 (10.5)	2 (6.1)	2 (13.2)	
Post-Transfusion 24th-hour ERCM/ Pre-Transfusion ERCM (%)	Med (IQR)	94.36 ± 12.67 (94.05)	90.45 ± 18.28 (88.13)	91.69 ± 18.1 (87.08)	0.227
	Under 85	7 (18.4)	12 (36.4)	3 (20)	0.255
	Range 85–115	30 (78.9)	18 (54.5)	11 (73.3)	
	Above 115	1 (2.6)	3 (9.1)	1 (6.7)	

IQR (Interquartile Range); ERCM (Estimated Red Blood Cell Mass).

**Table 4:** Comparison of Hb, Hct, and Plt Values in Metopic, Sagittal, and Coronal Groups at Pre-op and Post-op 1st and 24th hours.

	Metopic	Sagittal	Coronal	*p*-Value
Pre-op Hb (g/dL)	11.06 ± 1.17 (10.9)	11.06 ± 0.78 (11)	10.84 ± 1.12 (11)	0.789
Post-op 1st-hour Hb (g/dL)	10.25 ± 1.56 (10.1)	10.14 ± 1.19 (10.2)	10.36 ± 1.75 (9.7)	0.959
Post-op 24th-hour Hb (g/dL)	10.18 ± 1.14 (10.3)	9.9 ± 1.78 (9.7)	9.88 ± 1.68 (9.7)	0.411
Pre-op/Post-op 1st-hour Hb Pre-op/Post-op 24th-hour Hb	**0.002** **0.002**	**0.001** **0.001**	0.222 **0.020**	
Pre-op Hct (%)	32.12 ± 3.22 (31.55)	32.35 ± 2.46 (32.1)	32.03 ± 2.76 (32.6)	0.631
Post-op 1st-hour Hct (%)	29.88 ± 3.96 (30.2)	30.18 ± 3.34 (30)	30.93 ± 4.69 (30.5)	0.927
Post-op 24th-hour Hct (%)	30.08 ± 3.36 (30.3)	29.02 ± 4.98 (28.7)	29.13 ± 4.49 (28.9)	0.391
Pre-op/Post-op 1st-hour Hct Pre-op/Post-op 24th-hour Hct	**0.005** **0.007**	**0.001** **0.003**	0.233 **0.011**	

Hb (Hemoglobin); Hct (Hematocrit).

**Table 5:** A comparison of values of the Intra-op and total first 24 h CBL of the Metopic, Sagittal, and Coronal Groups; Transfusion ERCM; Transfusion PRBC; and Transfusion EBV.

	Metopic	Sagittal	Coronal
Intra-op CBL%	32.81 ± 14.08 (34.87)	31.81 ± 17.3 (33.66)	29.75 ± 15.63 (29.72)
Total First 24 h CBL%	45.94 ± 14.97 (45.96)	51.3 ± 23.78 (51.3)	47.21 ± 19.49 (52.25)
	0.000	0.000	0.005
Intra-op Transfusion ERCM %	26.46 ± 6.94 (27.14)	25.77 ± 7.69 (25.48)	27.03 ± 6.84 (25.66)
Total First 24 h Transfusion ERCM %	54.8 ± 16.98 (52.5)	56.68 ± 19.06 (56)	53.67 ± 16.08 (52.5)
	0.000	0.000	0.001
Intra-op Transfusion PRBC (mL/kg)	9.6 ± 2.39 (10)	9.5 ± 2.88 (9.52)	9.81 ± 2.24 (10)
Total First 24 h Transfusion PRBC (mL/kg)	14.57 ± 4.47 (13.33)	15.47 ± 5.22 (15)	14.11 ± 3.23 (14)
	0.000	0.000	0.004
Intra-op Transfusion EBV%	12 ± 2.99 (12.5)	11.87 ± 3.6 (11.9)	12.26 ± 2.8 (12.5)
Total First 24 h Transfusion EBV %	18.3 ± 5.41 (16.49)	19.19 ± 6.55 (18.15)	18.3 ± 3.87 (18.33)
Preop-postop	0.000	0.000	0.003

CBL (Calculated Blood Loss percentage); ERCM (Estimated Red Blood Cell Mass); PRBC (Packed Red Blood Cells); EBV (Estimated Blood Volume).

## Data Availability

The data supporting the findings of this study are available from the corresponding author upon reasonable request.

## Abbreviations

ASA	American Society of Anesthesiologists
CBL	Calculated Blood Loss
EACS	Endoscopy-Assisted Craniosynostosis Surgery
EBL	Estimated Blood Loss
EBV	Estimated Blood Volume
ERCM	Estimated Red Blood Cell Mass
Hb	Hemoglobin
Hct	Hematocrit
ICU	Intensive Care Unit
IQR	Interquartile Range
mL	Milliliters
mm^3^	Cubic Millimeter
OR	Operating Room
PACU	Post-Anesthesia Care Unit
PRBC	Packed Red Blood Cells
SD	Standard Deviation
µg/kg/min	Micrograms per Kilogram per Minute
mg/kg	Milligrams per Kilogram
min	Minutes
g/dL	Grams per Deciliter
